# Supplementary material for: Characterizing cell-type spatial relationships across length scales in spatially resolved omics data
Source: Nat Commun. 2025 Jan 3;16:350. doi: 10.1038/s41467-024-55700-1 (PMC11699133; doi:10.1038/s41467-024-55700-1)
Supplement: Supplementary file 3 — Supplementary Software 1 [file 41467_2024_55700_MOESM3_ESM.zip › CRAWDAD/plots/supplementary/S7_cluster_exp_PBVN_annot.pdf]

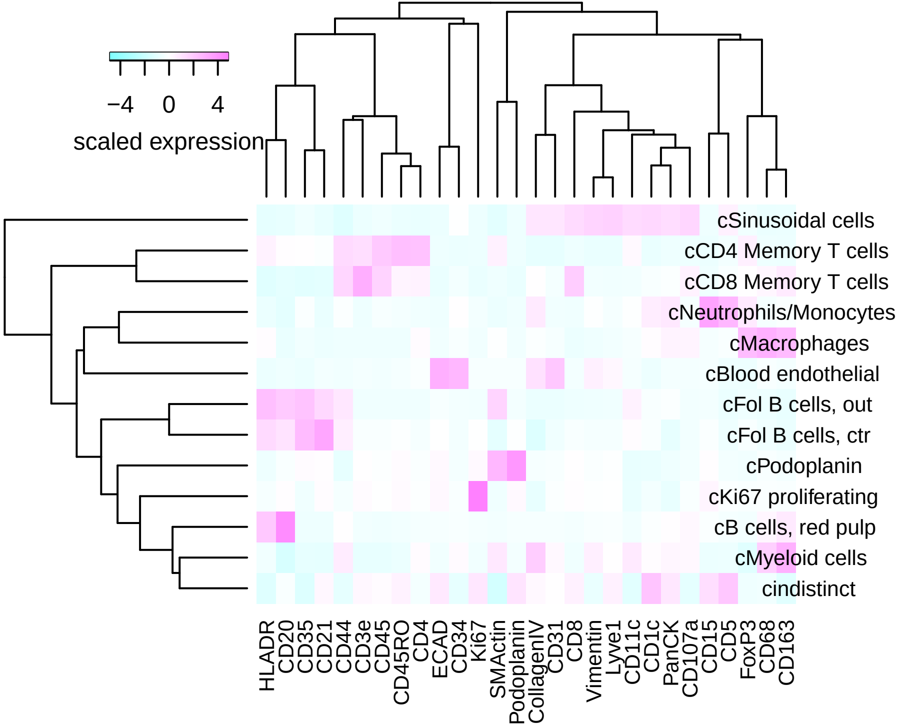
−4
0
4
scaled expression
cSinusoidal cells
cCD4 Memory T cells
cCD8 Memory T cells
cNeutrophils/Monocytes
cMacrophages
cBlood endothelial
cFol B cells, out
cFol B cells, ctr
cPodoplanin
cKi67 proliferating
cB cells, red pulp
cMyeloid cells
cindistinct
HLADR
CD20
CD35
CD21
CD44
CD3e
CD45
CD45RO
CD4
ECAD
CD34
Ki67
SMActin
Podoplanin
CollagenIV
CD31
CD8
Vimentin
Lyve1
CD11c
CD1c
PanCK
CD107a
CD15
CD5
FoxP3
CD68
CD163
